# Afghan Health Related Concerns Following the US Withdrawal: Results of a Survey Given *via* Social Media

**DOI:** 10.3389/fpubh.2022.905481

**Published:** 2022-07-14

**Authors:** Jaffer Shah, Asghar Shah, Ahmad Fahim Tokhi, Jordan Shedrow, Nicolas Hernandez, Joseph Varney, Pashton Qaderi, Seyed Javad Masoumi, Shohra Qaderi

**Affiliations:** ^1^Medical Research Center, Kateb University, Kabul, Afghanistan; ^2^Afghanistan National Charity Organization for Special Diseases, Kabul, Afghanistan; ^3^Division of Biology and Medicine, Brown University, Providence, RI, United States; ^4^Department of Stem Cell and Regenerative Medicine, Eskiseir Osmangazi University, Eskiseir, Turkey; ^5^American University of the Caribbean Medial School, Cupe Coy, Sint Maarten; ^6^Psychology and Educational Science Department, Balkh University, Balkh, Afghanistan; ^7^Department of Psychology, Faculty of Psychology and Education, University of Tehran, Tehran, Iran; ^8^School of Medicine, Shahid Beheshti University of Medical Sciences, Tehran, Iran

**Keywords:** Afghanistan, concerns, mental health, US withdrawal, social media

## Abstract

**Background:**

The United States Armed Forces completed their withdrawal from Afghanistan on August 30th, 2021, ending 20 years of war in Afghanistan. This rapid timeline from announcement to withdrawal and subsequent power transfer had profound consequences on the Afghan people, particularly in the domains of health and healthcare.

**Methods:**

On 15 September 2021, we posted an anonymous online cross-sectional survey on social media (Twitter, Facebook, and WhatsApp groups) to collect data about respondents from Afghanistan. Questions focused on COVID-19 symptoms, concerns, and individual care with a focus on changes related to the United States (US) withdrawal from Afghanistan. The form was composed of 17 questions which included multiple choice, single choice, and numeric options. All questions were optional including demographic data.

**Results:**

Our survey yielded 1,074 responses from the Farsi version and 572 responses from the Pashto version for a total of 1,646 responses. 1,286 (80%) of respondents were in Afghanistan at the time of survey submission. Concerning the US withdrawal from Afghanistan, 26% (412) respondents were extremely concerned and 12% (181) were moderately concerned. A majority of respondents report concerns regarding mental health due to the US withdrawal. 27% (418) report extreme concern, 12% (186) report moderate concern, and 15% (229) report a little concern. There is a significant difference in the proportions of concern (for US withdrawal generally, as well as physical and mental health) across gender. 49% of Female respondents report extreme concern regarding the US withdrawal compared to 22% of Male respondents (*P* < 0.001). With respect to physical health concerns 36% of Females report extreme concern compared to 16% of Males (*P* < 0.001). Finally on the mental health concerns, 54% of Females report extreme concern compared to 22% of Males (*P* < 0.001).

**Conclusion:**

The results from this survey are susceptible to the possibility of internal validity and/or external validity. However, we are accepting of those possibilities considering this survey wasn't designed to be bulletproof, but rather serve as a voice for those who can't be heard and to inform the public of the hardships occurring across the globe due to a steadfast retraction of the US footprint from their soil. Our findings indicate salient changes and public health concerns among Afghans following the US withdrawal from the region. These concerns varied across gender and ethnic groups. Our findings may serve as the first step in addressing the health concerns of Afghans following two decades of US military presence. The results should be understood through the limitations associated with a survey study design. Future research and policy aimed at tackling short and long-term health and social concerns in Afghanistan should consider the role of US withdrawal.

## Introduction

The United States Armed Forces completed their withdrawal from Afghanistan on August 30th, 2021, ending 20 years of war in Afghanistan (1). On July 8th, 2021, President Biden announced full troop withdrawal by August 31, 2021, and by August 15th, 2021, the Taliban entered Kabul (2). This rapid timeline from announcement to withdrawal and subsequent power transfer had profound consequences on the Afghan people, particularly in the domains of health and healthcare (3). The political situation, a direct result of US withdrawal, yielded public health consequences—healthcare workers fled, access to medicines was endangered, and the suspension of aid exacerbated existing humanitarian concerns (3). Another major public health concern is discontinuity of critical health services that were facilitated under a contract model between Afghanistan's Ministry of Public Health (MOPH) and NGOs (4).

These acute consequences should be understood with an understanding of the existing physical and mental afflictions present among individuals in Afghanistan. Due to living in a country experiencing decades long conflict, mental illness is prevalent in Afghanistan. A World Bank study from 2015 concluded that half of the Afghan population (aged 15 and older) is affected by either depression, anxiety, or post-traumatic stress disorder (5). Afghan women and girls experience a greater burden of mental illness due to limitations in access to care, stigma, and social restrictions (5). A 2015 study of Afghans in four Médecins Sans Frontières (MSF) hospitals based in Helmand, Kabul, Khost and Kunduz provinces found that 71.8% of patients experienced obstacles to access care and most patients had difficulties paying for healthcare (6). Moreover, patients reported violence, travel insecurity, and negative perceptions of care quality as barriers to seeking out care (6). Since Afghans from low socioeconomic status are more dependent on the health services provided by the public Basic Package of Health Services (BPHS) and the Essential Package of Hospital Services (EPHS), measures that sought to increase access to care and bridge healthcare disparities across social status, instability arising from US withdrawal may exacerbate existing inequities in healthcare access (7).

The US withdrawal influences the social determinants of health in Afghanistan, important factors that mediate all health outcomes. For example, a review on maternal health in Afghanistan concluded that health care, education, sociocultural practices, employment, income, food, and sanitation are all collectively critical determinants for maternal health (8). Given that the US withdrawal has influenced these factors it can be posited that new maternal health concerns may arise. An example of a social determinant of health is polluted air or water. One cross-sectional study of 27,565 Afghan mothers with under-five children in household found a 70.2% prevalence of solid fuel use, a 17.6% prevalence of acute respiratory infection (ARI) among children, and that children exposed to indoor air pollutants had higher prevalence of ARI compared to control. Aside from gendered and environmental determinants, racism and discrimination are also social determinants of health (9). Afghanistan is a diverse and multiethnic country, however reported ethnic disparities have been experienced by some in Afghanistan, and particularly by the Hazara minority (10). Unfortunately, there is a paucity of quantitative health data from Afghanistan that stratifies on ethnic identity; however, our study includes this measure. Finally, education quality and access are social determinants of health (9). Achieving parity in female education in Afghanistan has been a long-standing challenge, and further disruption, which may have resulted from US withdrawal, are barriers to women's health (11, 12). Given the culmination of mental health concerns, risk of violence, and gendered disparities acting in the Afghanistan context, a study assessing the public health concerns regarding US withdrawal is in order (13).

Despite bearing the direct public health consequences of a US withdrawal, the attitudes of Afghan people were not assessed in the decision-making process. This study is the first to investigate the public health and related social concerns of Afghans regarding the US withdrawal. We present the perspective of Afghans who have been affected by the US withdrawal. Using recent survey data, the aim of the present study was to assess these health concerns in a diverse population of Afghans from August through December of 2021.

## Methods

The primary goal of this cross-sectional study was to evaluate the public health and related social concerns regarding the US withdrawal from Afghan general population. In September 2020, we posted an online survey to a multitude of available social media platforms in the country (Twitter, Facebook, LinkedIn, and WhatsApp groups) to collect data about their symptoms, concerns, and individual action to take. Facebook posts were sharable to facilitate snowball sampling. This was an anonymous online survey that responders could choose to complete or not, and all the questions asked were optional; therefore, the number of respondents to each question varied. Minimal personal information was collected (only the information that may affect analyses of the data, such as ethnicity, age, and province). The distributed google form URL is composed of 17 questions including, multiple choices, single choice, and numeric. An online version of the survey was pilot tested with 20 participants who were excluded from the final results. Demographic data consist of age, sex, ethnicity, educational level, the geography of residence as well as current COVID-19 symptoms. Other questions asked participants about their concerns and lifestyle modification after the US withdrawal from the country. The survey was aimed at Afghan adults aged 18 and older, no other exclusion criteria were applied. After 6 weeks, we gathered 1,647 respondents and the data were analyzed by R.3.6.2 software.

## Results

Our survey yielded 1,074 responses from the Farsi version and 572 responses from the Pashto version for a total of 1,646 responses. 1,286 (80%) of respondents were in Afghanistan at the time of survey submission. Of this group, respondents hail from a diverse set of provinces in Afghanistan. The most common Afghan cities that respondents currently live in include Kabul 54% (694), Balkh 11.1% (146), and Herat 7.6% (98).

The median age of respondents was 28 (IQR 25, 33) and 224 (14%) were Female. The ethnicity of respondents was 39% Pashtun, 34% Tajik, 14% Hazara, 6.5% Other, 5.7% Uzbek, and 1.1% Turkmen. The education level of respondents varied from non-completion of high school to holding a doctorate. Most respondents (58%) held a bachelor's degree, 18% master's, 16% completion of high school, 4.0% less than high school, and 3.4% doctorate. Only 5.9% of respondents reported experiencing COVID-19 in the last 2 weeks.

Concerning the US withdrawal from Afghanistan, 26% (412) respondents were extremely concerned and 12% (181) were moderately concerned. With respect to concerns regarding physical health due to the US withdrawal, 19% (293) report being extremely concerned and 13% (203) report moderate concern. A majority of respondents report concerns regarding mental health due to the US withdrawal. 27% (418) report extreme concern, 12% (186) report moderate concern, and 15% (229) report a little concern. There is a significant difference in the proportions of concern (for US withdrawal generally, as well as physical and mental health) across gender. 49% of Female respondents report extreme concern regarding the US withdrawal compared to 22% of Male respondents (*P* < 0.001). With respect to physical health concerns 36% of Females report extreme concern compared to 16% of Males (*P* < 0.001). Finally on the mental health concerns, 54% of Females report extreme concern compared to 22% of Males (*P* < 0.001) (Table 1).

**Table 1:** Concerns about US withdrawal and physical and mental concerns due to US withdrawal.

**Concern**	**Not at all concerned**	**A little concerned**	**Moderately concerned**	**Very concerned**	**Extremely concerned**
US withdrawal	548 (34.9%)	197 (12.5%)	181 (11.5%)	232 (14.8%)	412 (26.2%)
Physical health concern	609 (39.1%)	211 (13.6%)	203 (13.0%)	241 (15.5%)	293 (18.8%)
Mental health concern	479 (30.5%)	229 (14.6%)	186 (11.9%)	257 (16.4%)	418 (26.6%)

With respect to the US withdrawal, 80% (1,285) of respondents report lifestyle changes since the US withdrew from Afghanistan. Among this group, 43.2% (555) report avoiding social gatherings, 34.8% (447) report stocking up on food and supplies, 34.9% (449) avoiding gym and/or exercise classes, and 34.7% (446) report not attending classes. 13.7% (176) of respondents report avoiding routine health care appointments. Respondents report several difficulties as having arisen after the US withdrawal. From most to least common: 54% report job loss, 42% report difficulty in accessing food, 37% report reduced wages or work hours, 33% report reduced hours of electricity per day, 32% report difficulty in accessing healthcare, 30% report transportation issues, and 24% report of issues related to childcare, access to wellbeing materials including cleaning supplies and sanitizer, and getting routine/essential medications. There was also a significant gendered difference in the reported difficulties arising from the US withdrawal. Specifically, 62% of Females reported job loss compared to 53% of Males (*P* = 0.012). 43% of Females reported difficulties in access to health care compared to 31% of Males (*P* < 0.001), and 30% of Females reported difficulties in access to routine medications compared to 24% of Males (*P* = 0.042). Other significant gendered disparities include childcare, access to wellbeing materials including cleaning supplies and sanitizer, transportation, and a reduction in daily hours of electricity (Tables 2, 3).

**Table 2:** Lifestyle changes since the US withdrew from Afghanistan.

**Characteristic**	**Hazara, *N* = 229**	**Other, *N* = 104**	**Pashtun, *N* = 617**	**Tajik, *N* = 542**	**Turkmen, *N* = 18**	**Uzbek, *N* = 91**
Avoiding social gatherings	115 (50%)	36 (35%)	211 (34%)	166 (31%)	4 (22%)	21 (23%)
Stocking up on food and supplies	80 (35%)	40 (38%)	131 (21%)	160 (30%)	5 (28%)	27 (30%)
Avoiding or canceling domestic travel	67 (29%)	21 (20%)	93 (15%)	105 (19%)	3 (17%)	21 (23%)
Working from home	55 (24%)	20 (19%)	79 (13%)	88 (16%)	3 (17%)	17 (19%)
Avoiding gym and/or exercise classes	114 (50%)	37 (36%)	114 (18%)	154 (28%)	2 (11%)	26 (29%)
Avoiding or canceling international travel	64 (28%)	22 (21%)	98 (16%)	98 (18%)	5 (28%)	15 (16%)
Avoiding routine health care appointments	54 (24%)	12 (12%)	45 (7.3%)	51 (9.4%)	2 (11%)	9 (9.9%)
Not attending classes *n* (%)	109 (48%)	37 (36%)	114 (18%)	159 (29%)	4 (22%)	21 (23%)

**Table 3:** Difficulties experienced following the US withdrawal.

**Characteristic**	**Hazara, *N* = 229**	**Other, *N* = 104**	**Pashtun, *N* = 617**	**Tajik, *N* = 542**	**Turkmen, *N* = 18**	**Uzbek, *N* = 91**
Reduced wages or work hours	103 (45%)	42 (40%)	238 (39%)	181 (33%)	5 (28%)	37 (41%)
I have lost my job	144 (63%)	54 (52%)	338 (55%)	288 (53%)	10 (56%)	44 (48%)
Childcare	87 (38%)	21 (20%)	145 (24%)	106 (20%)	3 (17%)	24 (26%)
Getting food	132 (58%)	48 (46%)	228 (37%)	228 (42%)	7 (39%)	39 (43%)
Getting cleaning supplies/hand sanitizer/PPE	82 (36%)	33 (32%)	116 (19%)	130 (24%)	3 (17%)	23 (25%)
Getting routine/essential medications	83 (36%)	25 (24%)	145 (24%)	126 (23%)	3 (17%)	20 (22%)
Transportation	113 (49%)	41 (39%)	108 (18%)	192 (35%)	4 (22%)	29 (32%)
Accessing health care	107 (47%)	34 (33%)	176 (29%)	179 (33%)	5 (28%)	26 (29%)
Reduced hours of electricity per day *n* (%)	101 (44%)	30 (29%)	168 (27%)	204 (38%)	4 (22%)	24 (26%)

Twenty-eight% (461) of respondents report being internally displaced following the US withdrawal. The most common Provinces of relocation include Kabul 44.5% (205), Balkh 12.6% (58), and Herat 5.9% (27). 16.6% (273) of respondents report becoming refugees following the US withdrawal. Among these respondents, the most common countries of relocation include Iran 39.6% (108) Pakistan 22.7% (62), and Germany 18.7% (51). Respondents rated their neighborhood as a place to live on a Likert scale from excellent to very poor. 34% (529) rated their neighborhood as average, 20% (310) as poor, 18% (280) as very poor, 14% (223) as excellent, and 13% (211) as good. When comparing whether their neighborhood has gotten better or worse compared to the same time last year, 42% (644) report their neighborhood as being much worse. 24% (369) report the neighborhood as being somewhat worse, 13% (197) somewhat better, 11% (166) much better, and 9.9% (153) the same.

## Discussion

In this study, we present the first data of Afghan public health concerns regarding the US withdrawal. A major finding in this survey conveys an array of variable concern related to individuals' physical and mental health. When considering physical health, 32% of the individuals displayed some level of concern ranging from moderate to extreme while an evaluation of mental health status reveals 54% of the group feeling some level of concern, ranging from little to extreme.

In 2011, Sayed (14) published an article to bring awareness to mental health disorders and how they constitute a major role amongst the total burden of disease on a global scale, further amplified by additive effects of early onset, heightened prevalence and persistence, and to a much greater extent, its relevance to conflict and post-conflict areas (p.6). He denotes a communal understanding by all that Afghanistan faces immensely high rates of mental health disorders in comparison to the outside world. This is a country shaped by the immersion of deep social trauma, an unwavering 30 plus years of armed conflict, modern warfare, natural disasters, substantial unemployment and poverty rates, the dismantling of social capital and scarce access to, or lack of, mental health services. According to Cardozo et al. (15), mental health facilities in Afghanistan are bleak and barren, if at all still standing. In Kabul, the main psychiatric hospital became collateral damage to the recent war. Three of the four of their community mental health centers are no longer functioning. This declination of brick-and-mortar health facilities is further exacerbated by a shortage of mental health care professionals that have proper training in Afghanistan, as similarly seen in Iraq after the US invasion (5).

In this study, females expressed more concerns than men which is in congruence with other studies in Afghanistan (16). To date, women report a greater tendency to worry than men and various theories have attempted to explain the gender differences (9). According to one theory, both men and women identify worry as a feminine gender role (17), other possibilities behind this theory are the role that a woman plays in society specific lower-status position in workplace or in the home. Meanwhile women are more prone to internalizing their problems. Despite all these gender-based factors, Afghan women are suffering from a broader spectrum of community structure and cultural barriers and most importantly, previous experiences from the first government of the Taliban and restrictive rules for Afghan women raise an extreme concern for Afghan women (14, 18).

Silove et al. (19) emphasized upon the strong relationship that exists between refugees and individuals exposed to mass violence and poverty in low-income countries (p.1). This financial burden places allocations of funds toward mental health services extremely low on the totem pole when compared to other necessities of daily living. The devolution of public expenditure in low-income countries renders tangible goods as of greater importance for the community. This hierarchal way of thinking severely constrains the ability to enhance overall measures for mental and physical health. Ekbald et al. (20) shares a commonality with Silove regarding the relation between warfare in low-income countries that lack a sound infrastructure, necessary skill sets and appropriate communal services. Ekbald et al. (20) further supports the understanding that mental health services receive little priority in impoverished settings, even though mass violence negatively impacts mental health in a multifactorial manner. Traumatic stress and grief-related psychological disorders constitute a direct impact from mass violence close to home. Pre-existing mental disturbances are nullified to betterment and destined to worsen as social support places rapidly decline in mass violence settings secondary to destruction or that it's no longer considered a safe place to be. As well, the long-term effects of armed conflict and mass violence prolong the opposition to communal growth and mental patients are often undermined, subjected to neglect, often resulting in no treatment (p.3).

80% of the individuals in this study reported to have experienced some variety of lifestyle change(s) after the withdrawal of US troops. Many authors have investigated the clinical relevance around major lifestyle changes and how each change directly effects and leads to another, moving in a pinball-like fashion. Islam et al. (21) published a paper in 2022 which further explores the interpersonal influences that major lifestyle changes have amongst one another to better understand the linear progression toward widespread hunger in Afghanistan (p.22). Nic et al. (6) focuses on the lifestyle changes of Afghanistan citizens leading to a deficit in their access to appropriate healthcare. The violence caused by political instability in Afghanistan is a direct barrier to healthcare for many. Active combat along roads prevents citizens from accessing clinics (p.5). Informational surveys like this one could help yield a better understanding of this linear progression to best understand how to mitigate this course and alter the trajectory from a hungry and sick community to a plan that works. For example, understanding that loss of employment leads to a reduced household income which leads to less food on the table.

An important aspect of the US withdrawal from Afghanistan was the financial support that was simultaneously discontinued at this time. Dobbins et al. (22) explains how the loss of financial support can crumble a country that already lives in poverty. The focus of their study highlights the city of Kabul and how it will become a much heavier contested city due to the US withdrawal (p.8).

The third major finding of this survey reveals the withdrawal of US troops has catalyzed internal displacement of Afghan citizens, ultimately leading to some individuals becoming refugees. It's important to understand that individuals who were not forced to relocate still endured impactful changes to their existing neighborhood conditions. This study revealed that 44% of the individuals had to relocate to a new home, whether internally or crossing borders into another country (23, 24).

To be forced to leave your home is immensely painful and a large endeavor. Unfortunately, some individuals don't know where or how to find a new home and endure a plethora of new problems once they get there. Kheradyar and Couch (10) published an article in 2019 that outlines many of the new problems they face after relocating, such as adapting to a new culture and/or language, discrimination they did not face before, employment issues, and service access (p.8). In another study from 2021, Mantoo breaks down the financial needs of internally displaced people in Afghanistan (p.3). UNHCR's Supplementary Appeal for the Afghanistan Situation estimates that $62.8 million in U.S. dollars is still needed for these internally displaced individuals. This amount remains labeled as “critical needs” (23, 24).

The situation can be alleviated only by international aid and engagement. Collective international efforts to address and face short-term and long-term humanitarian and healthcare challenges are imperative.

Limitation: Due to a deep gap between male to female literacy in Afghanistan, a higher number of males are using mobile phones than women, as shown in this study the number of women is under-represented (14%) which is a significant limitation and it should be considered in further studies. The findings of this study are limited and not generalizable due to the convenience sample nature of the participants. In addition to women, certain ethnic minorities were underrepresented among the respondents.

In conclusion: This survey was created to provide preliminary data that can be useful in understanding the scope and scale of the problems people Afghans are facing secondary to the withdrawal of US troops from Afghanistan. We hope this survey can provide the framework for future studies and inform health and foreign policy experts involved in the region.

## Data Availability Statement

The original contributions presented in the study are included in the article/supplementary material, further inquiries can be directed to the corresponding author/s.

## Author Contributions

JShe, AS, and SQ conceived and designed the study and wrote the manuscript. JSha, AFT, and NH helped collect data. JV, JShe, and SJM performed the statistical analysis and wrote the manuscript. PQ and JShe confirmed the eligibility of the participants' for the study and supervised the whole study and approved the final version of the manuscript. All authors contributed to the article and approved the submitted version.

## Conflict of Interest

The authors declare that the research was conducted in the absence of any commercial or financial relationships that could be construed as a potential conflict of interest.

## Publisher's Note

All claims expressed in this article are solely those of the authors and do not necessarily represent those of their affiliated organizations, or those of the publisher, the editors and the reviewers. Any product that may be evaluated in this article, or claim that may be made by its manufacturer, is not guaranteed or endorsed by the publisher.
